# Trajectories of affective disorders: neurobiological mechanisms during symptom change

**DOI:** 10.1007/s00115-025-01917-4

**Published:** 2025-11-20

**Authors:** Ulrich W. Ebner-Priemer, Judith Alferink, Michael Bauer, Udo Dannlowski, Irina Falkenberg, Andreas J. Forstner, Tim Hahn, Markus Junghöfer, Tilo Kircher, Luisa Klotz, Julia Martini, Eva Mennigen, Igor Nenadić, Carmine Pariante, Andrea Pfennig, Michael Ziller, Susanne Meinert

**Affiliations:** 1 Karlsruher Institut für Technologie (KIT), Institute of Sports and Sports Science, Karlsruhe, Germany; 2 Department of Psychiatry, University of Münster, Münster, Germany; 3 Department of Psychiatry and Psychotherapy, Technische Universität Dresden, Dresden, Germany; 4 Institute for Translational Psychiatry, University of Münster, Albert-Schweitzer-Campus 1, A9a, 48149 Münster, Germany; 5 Department of Psychiatry and Psychotherapy, Philipps-Universität Marburg, Marburg, Germany; 6 Institute for Human Genetics, University of Bonn, School of Medicine & University Hospital Bonn, Bonn, Germany; 7 Institute for Biomagnetism and Biosignal Analysis, University of Münster, Münster, Germany; 8 Department of Neurology with Institute of Translational Neurology, University of Münster, Münster, Germany; 9 Institute for Translational Neuroscience, University of Münster, Münster, Germany; 10 Department of Psychiatry, Medical School and University Medical Center OWL, Protestant Hospital of the Bethel Foundation, Bielefeld University, Bielefeld, Germany; 11 Institute of Neuroscience and Medicine (INM-1), Research Center Jülich, Jülich, Germany; 12 Department of Psychiatry and Psychotherapy, Central Institute of Mental Health, University of Heidelberg, Medical Faculty Mannheim, Mannheim, Germany

**Keywords:** Biomarkers, Affective symptoms, Mood disorders, Risk factors, Digital monitoring, Biomarker, Affektive Symptome, Stimmungsstörungen, Risikofaktoren, Digitales Monitoring

## Abstract

Effective treatment of affective disorders (AD) requires a deep understanding of the underlying neurobiological mechanisms. However, in machine-learning-based analyses, cross-sectional studies have failed to identify robust individual-level biomarkers. Research Domain A of CRC/TRR393 addresses this gap by implementing longitudinal, multimodal studies using real-time mobile assessments. Central to this effort is the identification of “inflection signals”—clinically meaningful symptom changes marking transitions from euthymia to depressive or (hypo)manic episodes. These critical windows are captured through digital phenotyping and ecological momentary assessments and followed up by in-depth neurobiological profiling. Six projects examine the dynamic interplay of behavioral, cognitive–emotional, molecular, immune, and neural mechanisms during these transitions. Project A01 validates early-warning models using digital phenotypes and machine learning. Project A02 maps structural and functional brain changes in relation to disease course and risk factors. Project A03 investigates the role of microglial immune activation in recurrent depression. Project A04 investigates neurobiological alterations after inflection signals using intensive, multimodal data acquisition conducted both in laboratory settings and in the participants’ personal environments. Project A05 adds molecular and immunological profiling and integrates findings from human and animal data. Project A06 studies trajectories from bipolar at-risk states to full-blown illness. Together, these projects form the empirical foundation for mechanism-based interventions (Domain C) and theoretical modeling of symptom trajectories (Domain B).

## Abstract (de)

Um affektive Störungen (AD) wirksam behandeln zu können, ist ein Verständnis ihrer neurobiologischen Mechanismen unerlässlich. Querschnittliche Studien stoßen hier an methodische Grenzen: In Machine-Learning-Studien ließen sich keine verlässlichen individuellen Biomarker identifizieren. Domain A des CRC/TRR393 adressiert diese Lücke durch längsschnittliche, multimodale Studien mit innovativen mobilen Erhebungsverfahren. Zentral sind sog. Inflection-Signale („inflection signals“) – zeitlich klar identifizierbare symptomatische Veränderungen, die den Übergang von Euthymie zu affektiven Episoden markieren. Diese kritischen Zeitfenster werden per mobiler, digitaler Phänotypisierung und breiter neurobiologischer Erhebung in Echtzeit identifiziert und untersucht. In 6 Projekten werden diese Übergänge aus verhaltensbezogener, kognitiv-emotionaler, molekularer, immunologischer und neurobiologischer Perspektive analysiert. A01 entwickelt und validiert digitale Frühwarnmodelle zur Vorhersage von Episoden. A02 analysiert Hirnstruktur und -funktion im zeitlichen Verlauf und in Abhängigkeit von Risikofaktoren. A03 untersucht mikrogliäre Immunaktivierung als möglichen Mechanismus rezidivierender Depression. A04 charakterisiert neurobiologische Veränderungen nach Inflection-Signalen per intensiver, multimodaler Datenerhebung. A05 ergänzt molekulare und immunologische Daten bei denselben A04-Probanden und erweitert die Analysen um Tiermodelle. A06 untersucht Übergänge vom Risiko für eine bipolare Störung (Bipolar-at-Risk-Stadium) zur klinischen Manifestation. Die Arbeiten in Domain A bilden die empirische Grundlage für mechanismenbasierte Interventionen (Domain C) und eine vertiefte konzeptuelle Modellierung von Symptomverläufen (Domain B).

## Background

Recent large-scale studies employing sophisticated machine learning (ML) have failed to identify individual-level biomarkers for major depressive disorder (MDD; [13]), highlighting the limitations of conventional case-control designs and the need for longitudinal research approaches. Advances in mobile assessment technology have made it possible to (a) predict and prevent upcoming affective disorder (AD) episodes by detecting inflection signals and early warning signals in real time; (b) understand whether neurobiological mechanisms shift around inflection signals and whether these changes forecast clinical trajectories; and (c) investigate whether life events modify emotion regulation, expectation, social interaction, and behavioral rhythms, thereby shaping long-term illness courses.

Until recently, multiple methodological challenges have impeded progress in these areas. Chief among them is the dilemma of needing simultaneously high-resolution data (to detect changes in time) over prolonged assessment periods (as new episodes are rare), while minimizing participant burden (to ensure adherence; [2, 3]). To overcome these challenges, we have established a cutting-edge mobile research infrastructure to detect in real-time (a) manic and depressive symptoms, (b) severe life events (SLEs), (c) altered expectation, and (d) social interaction patterns.

**Fig. 1:**
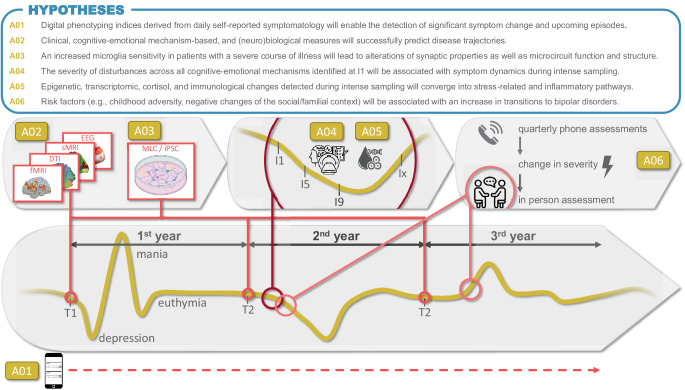
Overview of projects A01–A06. The six projects address different aspects of trajectory assessment, ranging from long-term to short-term intensive designs, from risk evaluation to prospective prediction, and integrate multimodal data collection with digital phenotyping in real-world settings. For each project, one central hypothesis is presented as an example to illustrate the planned analyses. Abbreviations: *I9/I5/I9/Ix* 8-week intensive weekly sampling with an additional follow-up assessment after remission (*Ix*), *DTI* diffusion tensor imaging, *EEG* electroencephalography, *fMRI* functional magnetic resonance imaging,* iPSC* induced pluripotent stem cells, *MLC* human microglia-like cells, *sMRI* structural magnetic resonance imaging, *T1/T2/T3* three yearly assessments

To summarize, six coordinated projects aim to delineate trajectories of symptom change and identify associated stressors, risk and resilience factors, and their behavioral, cognitive–emotional, and (neuro)biological mechanisms. Project *A01 *evaluates established digital phenotype models for early warning signs, investigating whether concepts such as “critical slowing down” and the “process control methods” can detect imminent AD episodes when based on appropriate data sets. We will also test whether cutting-edge ML approaches enable accurate forecasting of upcoming affective episodes and allow for predictions at the single-subject level. Project *A02 *will analyze brain imaging correlates of disease trajectories, associating brain structure and function with previous and prospective disease trajectories, risk and protective factors, as well as biological and behavioral measures from the other Domain A projects. It aims to ultimately contribute to predictive, individualized signatures for courses of illness. To this end, project *A03* leverages personalized in vitro disease models to test the hypothesis that microglia–neuron interactions are altered by the combination of genetic and environmental risk factors in MDD patients with low and high recurrence rates. Project *A04 *investigates short-term neurobiological changes following detected inflection signals through intensive, multimodal assessments over 8 weeks, including neuroimaging (magnetic resonance imaging [MRI], magnetoencephalography [MEG], electroencephalography [EEG]), biosampling (e.g., cortisol, blood, hair), and mobile assessments (e.g., EEG at home, Cardiowatch, Ecological Momentary Assessment). Project *A05 *includes the same subjects as *A04,* conducting complementary molecular and immunological analyses associated with symptom changes and illness courses, and will also perform transcriptomic and immunological characterization of selected animal models. Project *A06 *examines risk and resilience factors, symptom trajectories, and brain abnormalities to identify precursors and transition characteristics from at-risk stages to manifestation in individuals at risk of bipolar disorder (BD). Continuous mobile assessment will complement intensive sampling conducted around symptom changes both before and at the manifestation of the disorder (Fig. 1).

## A01—Longitudinal monitoring in affective disorders: real-time mobile assessment for early recognition of symptom changes

Continuous monitoring of digital phenotypes combined with real-time analyses has the potential to resolve core issues in AD research and treatment: the early detection, prediction, and (finally) prevention of symptom changes and new episodes. With the CRC/TRR393 acquiring the largest mobile assessment data set available worldwide, we will have, for the first time, enough upcoming episodes detected with a sufficiently high temporal precision to appropriately test existing theoretical models, such as “critical slowing down” and “process control methods,” and to develop ML-based individualized predictions of upcoming symptom changes and episodes. To achieve these goals, project *A01* has three work packages (WPs), using continuous, longitudinal data from the whole CRC/TRR393 cohort (*n* = 1500; from *S01, S02*): *WP1 *aims to evaluate established digital phenotype models for early warning signs by employing conventional inference statistical methods to ensure the comparability of our results with previous work; *WP2 *will examine smart digital phenotypes, as numerous existing ones currently lack substantial validity; and *WP3* will harness ML technology to facilitate personalized multivariate predictions. We will commence with the analyses in all WPs with existing digital phenotyping data from BipoLife and FOR2107 (ReMAP) and proceed with continuously acquired self-report and digital phenotyping data from the CRC/TRR393.

We aim to answer the following questions: Do existing models and theories on symptom dynamics, such as the “critical slowing down” and the “process control methods,” allow for the detection of significant symptom changes and upcoming episodes when based on appropriate data sets? Will the integration of smart digital phenotypes [12], such as those based on voice samples, improve the validity and eventually the detection of upcoming affective episodes? Will cutting-edge ML approaches enable accurate forecasting of upcoming affective episodes and allow for predictions at the single-subject level?

## A02—Longitudinal trajectories of brain changes as mediators of course of illness: intermittent sampling

Project *A02* investigates biopsychosocial mechanisms underlying disease trajectories across the lifespan through multilevel data integration. It is the analytical branch of S02 leveraging the rich intermittent sampling from the GEMCO cohort [11] to study risk/protective factors, their determinants, and neurobiological correlates, which remain poorly understood.

*A02* examines associations between brain structure/function and (a) prior disease course; (b) future trajectories, including inflection signals (S01); (c) early/late environmental factors; (d) biological markers (immunology, polygenic risk score, epigenetics, stress hormones); (e) behavioral measures (e.g., cognitive–emotional traits); and (f) treatment variables (medication, hospitalization). *A02* integrates longitudinal data from the FOR2107 [6] and Early-BipoLife [10] cohorts (baseline *n* = 4105, three follow-ups), CRC/TRR 393 time points (T1, T2), and mobile assessments (S01, S02, A01), totaling five measurement points over 11 years.

*WP1* applies clustering methods to identify clinical trajectories using clinical, psychopathological, psychosocial, and behavioral data. Illness course features (inflection signals, episodes) and variability will be modelled (Fig. 1). *WP2* links identified trajectories to MRI-based structural and functional brain changes, exploring how early and current risk/protective factors mediate or moderate associations. *WP3* (with S03) uses ML to predict future disease trajectories (e.g., hospitalization, functioning) and inflection signals. Predictors include neuroimaging, cognitive–emotional, genetic, immunological (with A05), and clinical data. *WP4* fuses multimodal data to assess each modality’s contribution to individual illness trajectories.

**Fig. 2:**
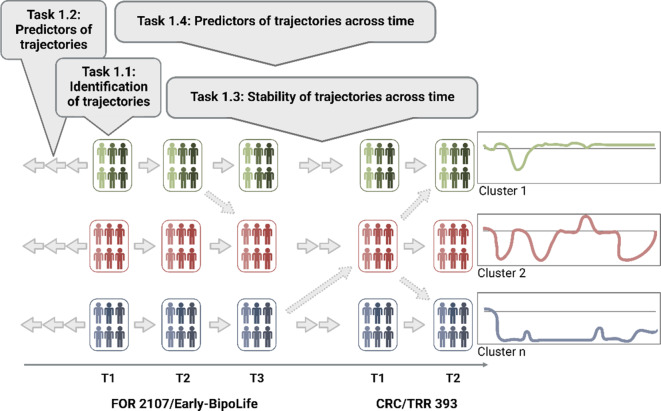
Retrospective, intermittent state (from time points of the GEMCO) and continuous data between time points will define clinical clusters. Resulting clusters and predictive variables will be integrated into neurobiologically informed *WPs* *2–4*. The latter will use intermittent MRI brain imaging data to characterize trajectories neurobiologically (*WP2*) and improve predictive power (*WPs* *3, 4*)

The longitudinal, integrative approach of *A02* will provide novel insights into complex interactions shaping disease trajectories and inform personalized prevention and intervention strategies ([7]; Fig. 2).

## A03—Dissecting the contribution of immune cell activation-dependent neuronal connectivity changes to disease trajectories in MDD

Affective disorders are strongly associated with altered inflammatory responses, affecting the innate immune system, including monocytes and microglial cells. However, it is unknown how these neuroinflammatory processes contribute to the trajectories of AD. *A03 *will test the hypothesis that increased (neuro-)inflammatory processes in patients with severe depression arise from the increased activation or sensitivity of immune cells of the central nervous system, resulting in the modulation of neuronal circuit function. To this end, *A03* will compare two extreme groups of MDD patients from the GEMCO cohort, namely, individuals with high recurrence rates and a high joint genetic and environmental risk factor load (*n* = 20) as well as patients with low recurrence and low risk factor load (*n* = 20). Specifically, *A03* will (a) determine whether the patient group with a severe course of illness exhibits an increased sensitivity and activation state of microglial cells; (b) evaluate whether the sensitivity or activation state of microglial cells in MDD is associated with alterations in brain structure; (c) determine the functional impact of an altered microglia sensitivity/activation state on synaptic plasticity and microcircuit function and structure in vitro; (d) identify the underlying molecular and cellular mechanisms of altered microglia activation and microglia–neuron interaction; and (e) determine whether depressive episodes are preceded and/or accompanied by enhanced innate immune activation in the central nervous system (CNS).

We aim to answer the following questions: Does the combined impact of environmental and genetic risk factors in severe MDD contribute to increased activation/sensitivity of microglial cells in the CNS? Does the activation of CNS innate immune processes modulate neuronal circuit function in a patient group-specific manner? Is this modulation altered during depressive episodes through epigenetic or transcriptomic mechanisms induced during/before the episode?

## A04—Brain network characterization of symptom changes using intensive sampling

Timely identification of prodromal transition phases—typically lasting 1–2 months and marked by subthreshold symptoms—is critical for developing predictive models and understanding cognitive–emotional mechanisms underlying episode onset. *A04* targets this clinically actionable window by providing a systems neuroscience characterization of short-term symptom trajectories through dense, multimodal sampling. Thus, it complements the long-term perspective of *A02 *[1, 4], with a high-resolution short-term approach.

We will follow up 200 participants from GEMCO (*n* = 100 MDD, *n* = 50 BD, *n* = 50 healthy controls), beginning immediately after an inflection signal is detected. Participants undergo an intensive 8‑week protocol (I1–I9), or continue assessment until symptom remission (Ix), with weekly home-based assessments (I2–I4, I6–I8) and in-lab sessions at I1, I5, and I9. For patients with a depressive episode, a fourth in-lab session (Ix) will be conducted following remission.

*A04* pursues three aims: (a) *WP1:* Longitudinal assessment of system neuroscience parameters (MRI, EEG, MEG, behavioral, peripheral, and clinical measures) shortly after the inflection signal, after 8 weeks, and post-remission (if applicable), focusing on four cognitive–emotional mechanisms predictive of clinical outcomes. (b) *WP2:* High-resolution, home-based mobile assessments (behavioral, peripheral physiology, mobile EEG) over 8 weeks to map short-term symptom dynamics. (c) *WP3:* Integration of stationary and mobile data using ML to develop individualized models predicting short-term illness trajectories.

**Fig. 3:**
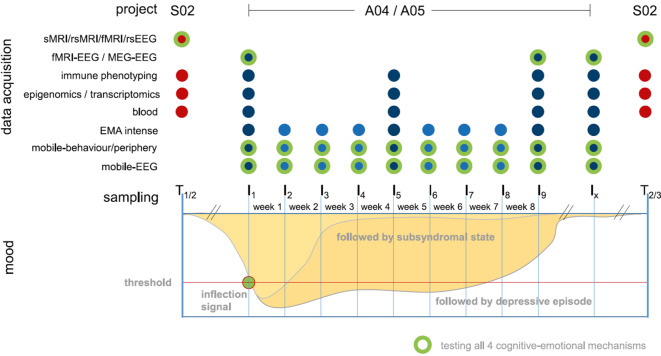
Study design of *A04 *and *A05*. Dark blue = lab-based data; light blue = mobile home-based data; red = intermittent sampling of *S02*; green = four cognitive–emotional mechanisms. *EEG* electroencephalography, *fMRI* functional magnetic resonance imaging, *MEG* magnetoencephalography, *sMRI* structural magnetic resonance imaging

By linking real-time symptom fluctuations to alterations in brain network function, *A04 *advances mechanism-based prediction and lays the foundation for future personalization in the prevention and treatment of AD (Fig. 3).

## A05—Molecular and immunological characterization of symptom changes and course of illness using intensive sampling

Previous studies have shown that ADs are multifactorial diseases [5]. However, the relationship of (epi-)genetic, transcriptomic, and immunological factors with symptom changes and illness courses in AD remains largely unknown. The aim of *A05* is thus to systematically characterize the link between molecular and immunological factors and (a) symptom changes and course of illness as well as (b) specific cognitive–emotional mechanisms. Using genotype data, Polygenic risk scores will be calculated for all GEMCO individuals (*n* = 1500) and tested for their association with illness courses. Epigenetic, transcriptomic, cortisol, and immunological analyses will be performed with 250 participants (*n* = 100 MDD, *n* = 50 BD, *n* = 100 healthy controls), of whom 200 (identical to *A04*) will be subjected to intensive sampling. Longitudinal molecular and immunological analyses will be performed at three time points after an inflection signal (I1, I5, I9, Fig. 3) and at remission if depressive symptoms persist after 8 weeks. Alterations will be associated with symptom changes, illness courses, and cognitive–emotional mechanisms. In addition, we will also perform transcriptomic and immunological analyses in selected animal models of the CRC/TRR393 to identify signatures related to disease course shared across species and investigate whether peripheral transcriptomic signatures can be used to infer changes in brain regions not accessible in humans. Integrative analyses of molecular and immunological data will be conducted to characterize multimodal signatures. Outcomes will be compared with *A03* and provided to other CRC/TRR393 subprojects. The results from *A05* will provide insights not captured by other assessment methods. They represent a key component for characterizing disease trajectories; they could lead to the identification of predictive factors of affective episodes; and they may serve as targets for new preventive, diagnostic, and therapeutic approaches.

## A06—Enhancing symptom monitoring in early affective disorders: exploring the trajectory from bipolar at-risk to bipolar disorder through intensive sampling

Often, BD emerges in early adulthood, preceded by an at-risk phase marked by subthreshold affective symptoms that can last months or years before the first full episode. Early identification during this stage is crucial but challenging due to the diversity of risk factors and the heterogeneity of clinical presentations. Building on our previous work in the BipoLife consortium [8, 9], we will assess precursors, clinical risk and resilience profiles, changes in social context, and brain abnormalities in more detail during transitions from at-risk stages to manifest BD. Overall, 150 at-risk participants, mainly from the FOR2107 and Early-BipoLife cohorts, will be followed up for 2 years using a multimodal, longitudinal approach and employing intermittent, continuous, and intensive sampling methods. In order to include a sufficient number of young individuals at risk for BD, additional participants will be newsly recruited. In addition to repeated clinical assessments of risk and resilience as well as early symptomatology, behavioral and chronobiological parameters will be continuously measured via mobile assessment (in cooperation with *B07*). Brain abnormalities will be assessed using fMRI (see *S02*), and immunological as well as (epi)genetic biomarkers will be evaluated through blood analysis (in cooperation with *A05*). For intensive sampling, participants will be invited to *A06* immediately after an inflection signal to verify the incident episode and to enable timely assessment of triggering risk and resilience factor as well as changes in the social context surrounding symptom changes or episodes. With this design, trajectories of early depressive and (hypo-)manic episodes will be described with unprecedented accuracy and reliability.

**Fig. 4:**
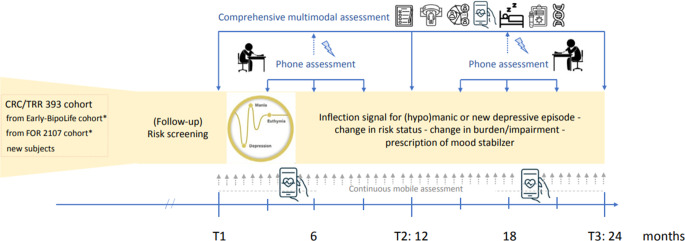
Participant flow, study design, and outcomes of *A06*. Asterisk 16–29-year-olds, no manifestation of bipolar disorder, schizoaffective disorder, or schizophrenia until new baseline (T1)

We aim to answer the following questions: How do clinical, behavioral, psychosocial, biological, and risk and/or resilience factors develop before, during, and after symptom (severity) changes prior to the onset of BD? How can patterns of psychopathology, behavior, and brain structure and function be used to predict symptom trajectories (Fig. 4)?

## Practical conclusion

- Early warning signals of affective episodes can increasingly be detected in real time.
- Mobile technologies enable continuous monitoring of symptoms, behavior, and physiology in everyday life.
- Intensive sampling allows for targeted investigations of neurobiological, molecular, and immunological changes during symptom transitions.
- Machine learning models based on multimodal data hold promise for individualized course prediction.
- Combining digital monitoring, neuroimaging, and molecular analyses opens new avenues for the development of new personalized preventive, diagnostic, and therapeutic approaches.

## Declarations

For this article no studies with human participants or animals were performed by any of the authors. All studies mentioned were in accordance with the ethical standards indicated in each case.
